# Isolation, Characterization and Biological Evaluation of Collagen from *Rhizostoma pulmo* Jellyfish from the Sea of Azov for Biomedical Applications

**DOI:** 10.3390/md24030109

**Published:** 2026-03-13

**Authors:** Oleg Kit, Sergey Golovin, Evgeniya Kirichenko, Alina Sereda, Yulia Gordeeva, Evgeniy Sadyrin, Andrey Nikolaev, Pavel Antipov, Aleksandr Logvinov, Maria Kaplya, Magomed Abdulkadyrov, Stanislav Rodkin

**Affiliations:** 1 Federal State Budgetary Institution “National Medical Research Center of Oncology” Russia, 14th Line Street, 63, Rostov-on-Don 344037, Russia; 2 Research Laboratory “Medical Digital Images Based on the Basic Model”, Department of Bioengineering, Institute of Living Systems, Don State Technical University, Rostov-on-Don 344000, Russiaalina.sereda2001@mail.ru (A.S.); gordeevayp@yandex.ru (Y.G.); heeelkaa@gmail.com (M.A.); rodkin_stas@mail.ru (S.R.); 3 Scientific and Educational Center “Materials”, Don State Technical University, Rostov-on-Don 344000, Russiaandreynicolaev@eurosites.ru (A.N.); sly_fox_03@mail.ru (P.A.); 4 Laboratory of Molecular Neuroscience, Research-Scientific Institute of Physical & Organic Chemistry, Southern Federal University, 194/2 Stachky Ave., Rostov-on-Don 344090, Russia

**Keywords:** collagen, jellyfish, *Rhizostoma pulmo*, biocompatibility, cytotoxicity, scaffolds, biomaterials

## Abstract

Collagen is a major extracellular-matrix protein widely used in regenerative medicine, yet conventional terrestrial sources raise biosafety and acceptability concerns, motivating the search for marine alternatives. This study evaluates the jellyfish *Rhizostoma pulmo* (*R. pulmo*) from the Azov Sea as a sustainable collagen source and assesses its suitability for biomedical materials. Acid-soluble collagen was extracted using 0.5 M acetic acid and purified by salt precipitation and dialysis, followed by physicochemical/structural characterization (sodium dodecyl sulfate–polyacrylamide gel electrophoresis (SDS–PAGE), Limulus amebocyte lysate (LAL) endotoxin testing, transmission electron microscopy (TEM), and immunofluorescence with type I collagen antibodies) and biological evaluation *in vitro* (3-(4,5-dimethylthiazol-2-yl)-2,5-diphenyltetrazolium bromide (MTT) cytotoxicity on MRC5 fibroblasts; adhesion and proliferation assays on HeLa cells). The extracted collagen showed a high yield (~26.2%), a type I-like electrophoretic profile with α-, β-, and γ-components, fibrillar ultrastructure by TEM, and positive type I collagen immunoreactivity; endotoxin levels were low (0.461 EU/µL), and no cytotoxicity was detected under the tested conditions. Porous collagen sponges/scaffolds were fabricated by lyophilization, displaying interconnected pores with an average size of ~80 µm and pH-dependent swelling, and they supported 3D cell growth and tumor-cell dissemination in an *in vitro* breast carcinoma scaffold model. Overall, Azov Sea *R. pulmo* collagen demonstrates promising structural quality, low endotoxin burden, and cytocompatibility, supporting its potential as a marine biomaterial for sponge/scaffold-based tissue engineering and wound-related applications.

## 1. Introduction

Collagen is one of the most abundant proteins of animal origin and a fundamental component of the extracellular matrix that determines tissue architecture and regulates key cellular processes, including adhesion, migration, and proliferation. Consequently, collagen-based materials are widely applied in regenerative medicine, pharmaceutics, and cosmetology. In clinical and preclinical studies, collagen is utilized both as a biomaterial and as a source of bioactive peptides, attributed to its biocompatibility, capacity to form fibrous/porous matrices, and participation in wound-healing mechanisms [1]. However, traditional collagen sources (primarily terrestrial animals) are associated with limitations, including zoonotic safety concerns and sociocultural acceptability issues, which have intensified interest in alternative bioresources, particularly marine sources [2].

Marine collagen is regarded as a promising raw material for the development of next-generation biopolymers: it can be processed into hydrogels, sponges, films, and composite matrices for delivery systems, tissue engineering, and wound dressings [1]. Marine invertebrates, including jellyfish, occupy a substantial niche in this field, as their biomass is significant in many regions, and mesogleal collagen demonstrates suitability for soft biomaterial formation while potentially mitigating risks associated with terrestrial sources [3]. Specifically, marine collagen from jellyfish offers reduced risk of prion and zoonotic viral transmission, absence of religious or sociocultural acceptability barriers, and simplified purification owing to the low content of lipids, pigments, and tightly crosslinked non-collagenous proteins in jellyfish mesoglea. In recent years, “jellyfish collagen” has emerged as a distinct branch of marine biopolymers, with accumulating data on its physicochemical properties, structural organization, and cell–matrix interactions that are critical for biomedical design [4].

The Sea of Azov has experienced sustained growth in both the abundance and biomass of scyphozoan jellyfish over many years, with associated ecological and socioeconomic consequences, as these organisms can significantly restructure trophic relationships, compete for food resources, and complicate fishing and recreational use of coastal waters [5,6]. Studies focused on the Azov basin emphasize that persistent seasonal aggregations of *R. pulmo* represent a substantial raw material resource, rendering “harvest–processing” strategies viable as a potential tool for simultaneously reducing ecological burden and generating high-value-added products [6,7,8]. In this context, the development of a technologically reproducible approach for collagen extraction and biomedical evaluation from Azov *R. pulmo* populations can be viewed as part of a broader concept of sustainable marine bioresource utilization, wherein an ecological challenge is transformed into a biomaterial source for medicine and related industries [3,8].

From a mechanistic perspective, the applied value of collagen is determined not only by its chemical composition but also by how cells recognize the matrix through receptor systems (integrins and heparan sulfate-mediated interactions), which influence cell adhesion, remodeling, and migration in wounds or tissue equivalents [2]. *R. pulmo* collagen (jellyfish collagen, JCol) has been shown to retain triple helical structure, form fibrillar assemblies, and display distinctive cell-adhesion characteristics compared with mammalian type I collagen [9]. Specifically, receptor-blocking experiments have revealed that JCol-mediated cell adhesion is primarily realized through β1-integrins, although the contribution of α2β1-integrin differs from that of mammalian type I collagen, and heparan sulfate/heparin binding may play a more prominent role for fibroblasts and mesenchymal stromal cells [10]. Such distinctions are important for bioengineering, as “fine-tuning” of the cellular matrix response often determines the success of wound dressings or tissue-engineered scaffolds [11,12,13].

In parallel, jellyfish collagen has been developed as a functional hemostatic material [14,15]. For *Rhopilema esculentum* collagen, highly porous sponges with superior water-absorbing capacity have been described; in rat hemorrhage models, such sponges demonstrated improved hemostatic performance compared to medical gauze, attributed to adhesion/activation of blood cells on the material surface and rapid clot formation [14,15]. Collectively, these findings support the concept of jellyfish collagen as a source of not only a “structural” biopolymer but also a functional platform for materials in which hemostasis and accelerated repair are key objectives [3,4].

Reportedly, *R. pulmo* collagen is characterized by an increased tryptophan content and exhibits antioxidant properties [16]. In addition to collagen, jellyfish tissue contains a range of other low-molecular-weight peptides with antioxidant activity [17].

Another rapidly growing application of marine collagen is cosmetology and anti-aging strategies, where emphasis is placed on bioactivities related to oxidative stress, photodamage, and dermal matrix degradation [18]. Reviews of marine collagen and peptides emphasize that potential anti-aging effects are attributed to a combination of antioxidant mechanisms, modulation of inflammatory responses, and maintenance of skin matrix homeostasis, as well as improved barrier and hydration characteristics with collagen-containing products [1,19,20]. Although the transition from “promising” effects to standardized claims requires reproducible protocols and comparable biomarkers, the trend toward marine collagen ingredients in cosmetic and biomedical applications is evident [20].

Despite the progress made, the field retains methodological and interpretational “gaps” that are critical for translation. Jellyfish collagen demonstrates inter-species and inter-population variability, and protocol differences (acid-soluble versus pepsin-soluble collagen, purification modes, crosslinking, sterilization) can alter structure and, consequently, cellular response [2]. The debate continues regarding which test batteries are sufficient for biomedical positioning—purely physicochemical/structural “identification” or mandatory extension to functional assays (cell adhesion, cytocompatibility, hemostasis, inflammatory markers) [3]. For several species, including *R. pulmo*, data linking structural parameters (triple helix retention, fibril genesis, interactions with glycosaminoglycans) to predictable biological outcomes remain needed, as these linkages determine material design for wounds and soft tissues [1,2,3].

The present study addresses *R. pulmo* as a source of marine collagen for biomedical applications, emphasizing reproducible extraction, comprehensive characterization, and biological evaluation. The aim was to isolate collagen from the jellyfish *R. pulmo*, inhabiting the Sea of Azov, perform comprehensive physicochemical and structural characterization, and evaluate key biological properties relevant to biomedical applications, including assessment of suitability as a basis for biomaterials, and demonstration of feasibility for creating collagen sponges and scaffolds with potential application in tissue engineering and regenerative medicine.

## 2. Results

### 2.1. Jellyfish Collagen Extraction

Acid-soluble collagen was successfully obtained from umbrella tissues of *R. pulmo* jellyfish collected in the Azov Sea. The final collagen yield after dehydration reached 26.2% ± 3.1% (*n* = 20), which is in good agreement with previously reported values for similar organisms [3]. Collagen isolated from mouse tails using the same extraction approach was used as a reference control at multiple stages of the study.

### 2.2. SDS-PAGE Analysis

**Figure 1:**
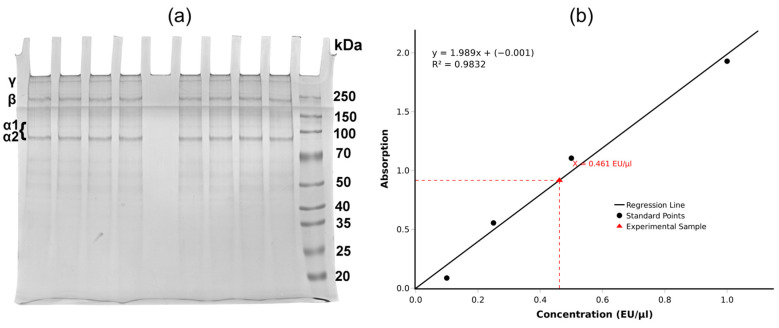
Structural investigation and bacterial endotoxin content test of jellyfish collagen: (**a**) SDS-PAGE of *R. pulmo* jellyfish collagen samples: on the side—molecular weight marker, γ—collagen α-chain trimer, β—collagen α-chain dimer, α1- and α2-collagen chains; (**b**) LAL test results of jellyfish collagen sample. The graph demonstrates a linear relationship between the concentration of standard samples and their absorbance at 405 nm. Black circles denote standard control points, red triangle—the position of the test sample on the curve. The regression equation and the coefficient of determination are displayed on the graph.

To analyze the structural characteristics of the isolated collagen from *R. pulmo* jellyfish, SDS-PAGE analysis was performed. As shown in Figure 1a, acid-soluble collagen extracts demonstrate a high degree of purity and the presence of distinct protein bands around 100 kDa, corresponding to α-chains, with additional bands of β- and γ-chains observed around 250 kDa, indicating dimeric and trimeric crosslinked forms, respectively, as well as a high-molecular-weight fraction located above the molecular weight marker. It is known that collagen consists of three α-chains, which cannot always be identified [21]. In addition, triple-helical proteins possess apparent electrophoretic mobility in SDS-PAGE, which may not always correlate with their molecular mass due to the low percentage of hydrophobic amino acid residues [22,23].

In our case, the first band is likely associated with the α-chain trimer (γ-component), and the second band with a molecular weight of about 250 kDa is the α-chain dimer (β-component) [24]. The third band, located around 95 kDa, can be interpreted as the α2-chain. At the same time, an extremely weak-intensity band with a molecular weight of about 105 kDa is visible, which may be associated with the α1-chain [24]. The very low staining intensity of this band may be explained by the fact that a considerable fraction of α1-chains is involved in intermolecular crosslinking and therefore present predominantly in β- and γ-components rather than in monomeric form. In addition, similar weak or barely detectable α1-chain bands have been reported in previously published SDS-PAGE profiles of collagen isolated from *R. pulmo*, suggesting that this feature may be intrinsic to jellyfish collagen rather than indicative of degradation. Importantly, no additional low-molecular-weight bands were observed, which confirms the absence of proteolytic fragmentation and supports the high purity of the sample. The obtained results are consistent with previous studies, which showed that SDS-PAGE for type I collagen typically reveals three bands that correspond to α-chains, as well as secondary collagen structures—β- and γ-components. It is reported that the molecular masses of α-chains range from 100 to 130 kDa, with β-component approximately 250 kDa [25].

It is also worth clarifying that a commercial mammalian type I collagen standard was not used for direct visual comparison, since the molecular masses of α-chains of collagen from invertebrates, such as jellyfish, systematically differ from those of mammalian collagen due to species-specific variations in the amino acid composition and molecular mass of collagen, as well as differences in post-translational modifications between vertebrate and invertebrate forms [18]. Therefore, the use of a mammalian standard for identification would be of limited accuracy and might not serve as an appropriate reference for this sample. Instead, we relied on direct comparison with published SDS-PAGE profiles obtained from the same biological source.

It should be noted that the obtained results correlate well with a recent study that analyzed the purity of lyophilized jellyfish collagen powder from *R. pulmo* presented by the company “Jellagen”. SDS-PAGE analysis of the collagen demonstrated bands in the α-range at 105 and 92 kDa and high-molecular-weight β (dimer) and γ (trimer) bands. At the same time, the band interpreted as the α1-chain had extremely weak intensity, as in our case, where it was practically not visible [26]. Also, in previous scientific works, it was shown that acid collagen extracted from jellyfish mainly represents type I collagen, consisting of 3 polypeptide chains: β-chain (~250 kDa), α1-chain (~140 kDa), and α2-chain (~100 kDa). However, there are other data in which the molecular mass of α1- and α2-chains of collagen isolated from jellyfish has higher values in SDS-PAGE [24].

Our data show that the collagen isolated from *R. pulmo* jellyfish can be classified as type I collagen or so-called type 0, as well as demonstrating high sample purity.

The SDS-PAGE profile of the isolated collagen demonstrates high sample purity, as evidenced by the absence of non-collagenous protein bands. Purity was further supported by the specificity of immunofluorescence labeling (Section 2.5) and low endotoxin burden (Section 2.3).

### 2.3. Endotoxin Determination

Each batch of obtained collagen was tested for bacterial endotoxin content by the LAL test method. The graph in Figure 1b demonstrates linearity, while the concentration of the test sample (0.461 EU/μL) is within the calibration range of standards (0.1–1.0 EU/μL) and corresponds to acceptable endotoxin content values in collagen products for biomedical application according to ISO 10993-11:2017 [27] and GOST ISO 10993-11-2021 [28]. The obtained endotoxin level indicates effective control of pyrogenic contamination during collagen isolation and processing. It should be noted that the LAL assay specifically detects bacterial endotoxins and does not account for non-endotoxin pyrogens. Overall, the results confirm that the studied collagen meets endotoxin-related safety requirements and can be considered suitable for further biological evaluation.

### 2.4. Transmission Electron Microscopy

**Figure 2:**
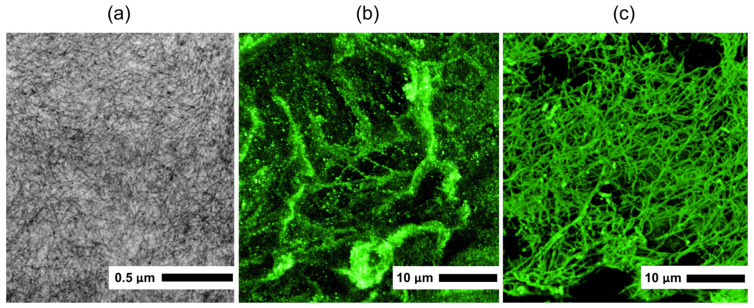
Microscopy of *R. pulmo* jellyfish collagen: (**a**) TEM of *R. pulmo* jellyfish collagen (scale bar: 0.5 μm); (**b**) immunofluorescence microscopy of *R. pulmo* jellyfish collagen (scale bar: 10 μm); (**c**) immunofluorescence microscopy of type I collagen from mouse tail (scale bar: 10 μm). The images are representative of collagen obtained in 5 independent extraction procedures.

Investigation by TEM of collagen from *R. pulmo* jellyfish showed that it has a fibrillar structure typical of native type I collagen [29,30]; collagen fibrils are visualized as osmiophilic fibers with a thickness of 5–15 nm (Figure 2a). The fibrils appear uniformly distributed and predominantly unbranched, forming loose bundles without evidence of aggregation or structural disruption, which is characteristic of well-preserved native collagen. No signs of denaturation or amorphous structures were observed, indicating that the collagen extraction procedure preserved the native ultrastructural organization of the fibrils. Denatured collagen would typically appear as amorphous, aggregated masses lacking discernible fibrillar periodicity and ordered bundle formation on micrographs [31].

### 2.5. Immunofluorescence Analysis

Immunofluorescence analysis (Figure 2b,c) demonstrated that antibodies to type I collagen were immunoreactive with collagen isolated from *R. pulmo* jellyfish as well as with mouse tail type I collagen used as a positive control, confirming the type I nature of the isolated jellyfish collagen. A distinct fibrillar fluorescence pattern was observed for the jellyfish collagen, similar to that of the positive control. No nonspecific staining or background fluorescence was detected, indicating the specificity of the antibody–antigen interaction.

### 2.6. Cytotoxicity Assessment Using MTT Assay

**Figure 3:**
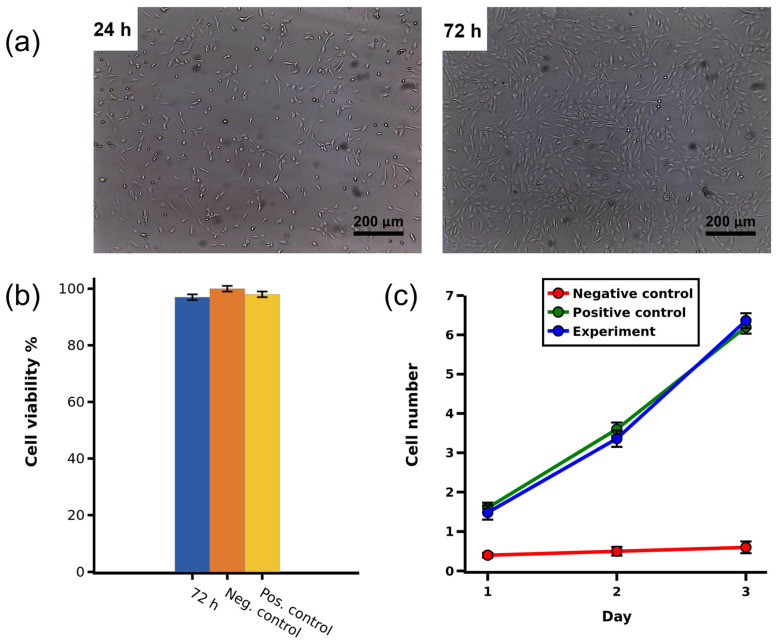
Assessment of cytotoxicity and cell adhesion of jellyfish collagen: (**a**) Adhesion of HeLa cells after 24 h and proliferation after 72 h of incubation (scale bar: 200 μm). (**b**) Viability of MRC5 cells with 0.5 mg/mL jellyfish collagen solution after 72 h incubation; 0.5 mg/mL solution of commercial type I collagen from rat tails was used as a positive control, whereas cells incubated in medium without collagen were used as a negative control. (**c**) Proliferation curves of HeLa cells over 72 h on different types of coatings; types of coatings: jellyfish collagen (experiment), mouse tail collagen (positive control), uncoated non-culture plastic (negative control). Values are presented as mean ± standard deviation (SD) for nine replicates (*n* = 9) in three independent experiments.

Cytotoxicity assessment was performed by the MTT test method with a culture of normal human fibroblast cells MRC5 in accordance with the ISO-10993-1 standard [32]. No significant difference was found between the cytotoxicity of collagen solutions compared to the control group in the MTT test after 72 h of cultivation (Figure 3). These results indicate acceptable cytocompatibility of the jellyfish collagen we obtained. Cytotoxicity screening of different concentrations of jellyfish collagen has been conducted in a large number of studies, and in general, their results are consistent and indicate the absence of cytotoxicity at concentrations similar to those used for mammalian collagen [3,24]. Based on this, we did not conduct screening but adopted a working concentration similar to the collagen concentration in a commercial type I collagen preparation from rat tails (1.2.001. LLC. BioloT, St. Petersburg, Russia) used for coating culture dish surfaces.

### 2.7. Cell Adhesion and Proliferation Analysis

Proliferation and spreading of HeLa cells on Petri dishes coated with jellyfish collagen and mouse tail collagen were quantified after their detachment using a Neubauer counting chamber. Figure 3b presents typical images of HeLa cell adhesion after 3 h and proliferation after 72 h of incubation. There was no significant difference between cell doubling time measured over 3 days on any collagen coating (Figure 3c). The degree of cell proliferation was also comparable on both coatings.

### 2.8. Production and Characterization of Collagen Sponges

**Figure 4:**
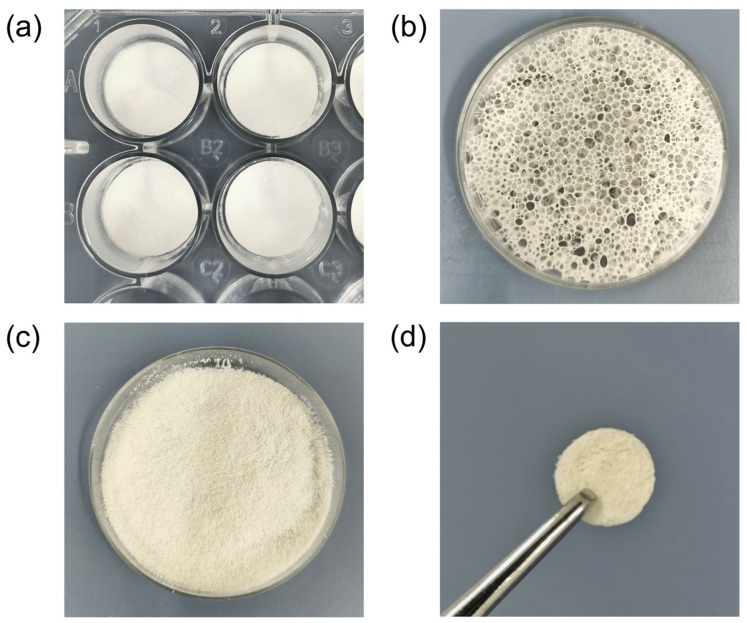
Appearance of three-dimensional scaffold constructs obtained from *R. pulmo* jellyfish collagen: (**a**) collagen films mounted in the wells of a culture plate; (**b**) a collagen scaffold; (**c**) a collagen sponge mounted in a Petri dish; (**d**) a subcutaneous collagen implant used in Section 2.9.

**Figure 5:**
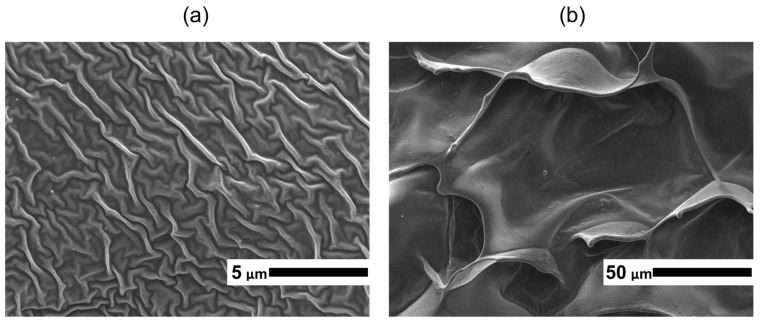
Characterization of collagen scaffolds using scanning electron microscopy. SEM images of the architecture of lyophilized jellyfish collagen scaffolds at (**a**) low (magnification 5×, accelerating voltage = 3.00 kV scale bar: 5 μm) and (**b**) high (magnification 500×, accelerating voltage = 6.00 kV, scale bar: 50 μm) magnification.

To assess the suitability of *R. pulmo* collagen as a raw material for the production of sponges and scaffolds for biomedical application, sponge-like scaffold constructs and scaffolds were manufactured, examples of which are shown in Figure 4. To obtain sponges approximately 3 mm high, collagen hydrogel with a concentration of 10 mg/mL was cooled to 4 °C, 15 mL of which was placed in a Petri dish (Ø = 60 mm) and frozen at −20 °C for 2 h and at −80 °C overnight, then dried in a freeze dryer. To obtain scaffolds, collagen hydrogel with a concentration of 4 mg/mL was cooled to 4 °C and dried in a freeze dryer for 24 h without freezing. The unfrozen gel, when evacuated in the drying vacuum chamber, turned into a fast-hardening foam, the porosity of which can be varied by the collagen concentration in the hydrogel. As a result, we obtained three-dimensional structures consisting of interconnected pores, the boundaries of which were determined by lamellar structures of dense afibrillar collagen (Figure 5a).

The scaffold pores, measured from SEM images, have an average pore size of 80 ± 6.2 μm. Thus, the average pore size in JCol scaffolds is within the range of pore sizes in mammalian collagen scaffolds, while the shape and uniformity of pores throughout the scaffold are comparable [33].

**Figure 6:**
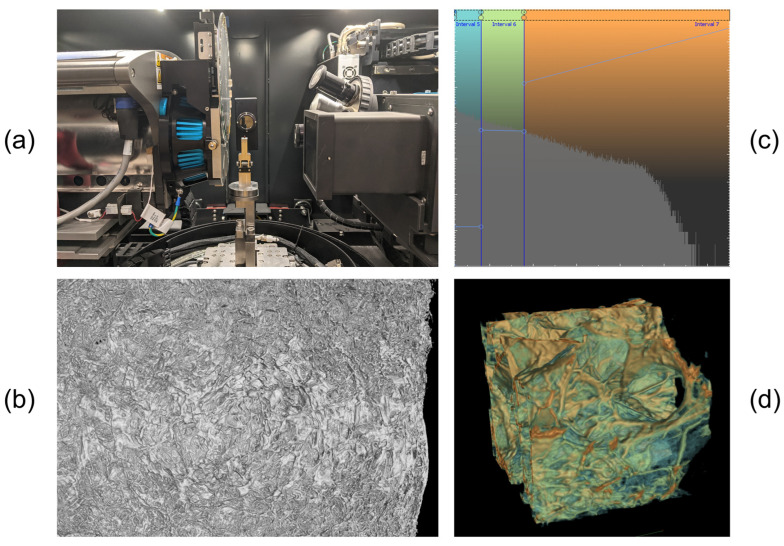
Microtomography of collagen sponges: (**a**) placement of the sample in the Xradia Versa 520 system; (**b**) general view of the surface of a collagen sponge; (**c**) histogram of the density distribution of the collagen sponge: regions with the lowest density are colored blue, denser ones—green, the densest—orange; (**d**) a sponge region measuring 0.35 × 0.33 × 0.34 mm, segmented using pseudocolor: the lowest-density areas are colored blue, denser areas are green, and the highest-density areas are orange.

Using microtomography, the density and homogeneity of the obtained scaffolds were assessed, evaluated by the distribution of iodine in the sample. Figure 6b shows the overall appearance of the sample surface. Then, a region measuring 0.35 × 0.33 × 0.34 mm was cut from the central segment, being the least susceptible to artifact formation during microtomography, and segmentation of this region was performed using pseudocolors (Figure 6d). Regions with the lowest density are colored blue, denser ones—green, the densest—orange (a scheme with a fragment of the density distribution histogram is presented in Figure 6c). Such a profile is typical for porous structures, where most of the frame consists of pore voids with low intensity, and the useful signal is represented by relatively small but uniformly bright objects representing cell walls.

### 2.9. Swelling and Stability

**Figure 7:**
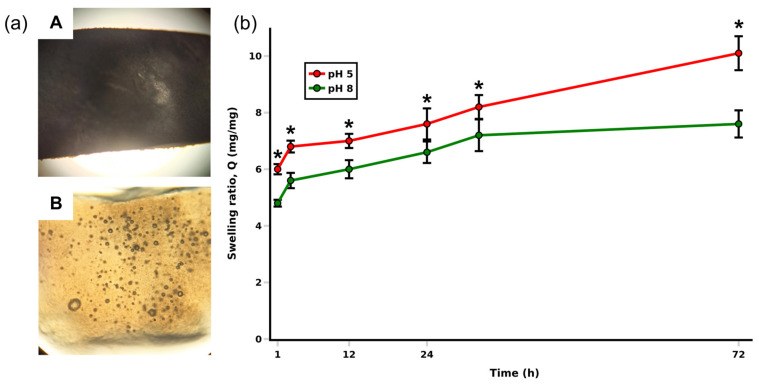
Study of the swelling behavior and stability of collagen sponges: (**a**) lyophilized (A) and hydrated (B) sponges; (**b**) changes in the swelling of collagen sponges over time in PBS over 72 h at pH 5.0 and pH 8.0. Q (swelling capacity) is expressed in mg/mg ± standard error of the mean, * *p* ≤ 0.05.

Collagen sponges for wounds should absorb exudate while maintaining moisture, which is extremely important for adequate tissue regeneration. Biomaterials with high swelling capacity and prolonged moisture retention protect granulation tissue and promote faster cell proliferation, preventing scar formation [34]. We conducted tests on the swelling capacity of collagen sponges. Figure 7 shows graphs of the swelling capacity of collagen sponges at pH 5.0 and pH 8.0. All scaffolds swelled rapidly during the first hour. At pH 5.0, the swelling coefficient Q was higher than at pH 8.0.

At pH 8.0, the equilibrium swelling of collagen was less than at pH 5.0, which can be explained by the acid solubility of collagen. But in general, collagen remains stable over the tested pH range (pH 5.0–8.0), which encompasses the relevant wound microenvironment pH values. In the investigated pH range (pH 5.0–8.0), the collagen-based material showed stable behavior without signs of abrupt destabilization; however, the equilibrium swelling at pH 8.0 was lower than at pH 5.0, which is consistent with the expected pH dependence of hydration in collagen-containing matrices. This direction of the effect agrees with the observations by Coppola et al., who reported that the swelling behavior of chitosan/collagen (CS/COL) scaffolds was generally higher under acidic conditions (pH 5.0) than at pH 8.0, and attributed these differences to pH-dependent ionization/solubility of the matrix components (in their system, primarily chitosan at pH values below its pKa), resulting in changes in water uptake and swelling ratio *Q* [35]. Similar trends have been described for collagen hydrogels: Setoyama et al. demonstrated pronounced pH-responsive swelling, with increased swelling under more acidic conditions and reduced swelling at pH above approximately 6, which they related to changes in the ionization state of functional groups and the solubility/interactions within the collagen network [36]. Therefore, the lower equilibrium *Q* at pH 8.0 compared with pH 5.0 observed in our study should be regarded as a reproducible and literature-consistent pattern rather than an anomaly, reflecting the general concept that pH modulates hydration and water retention via the acid–base properties of biopolymer matrices. Importantly, the tested pH interval is highly relevant to wound microenvironments. As discussed by Jones et al., the pH of healthy skin is typically mildly acidic (approximately 4.0–6.0), whereas chronic wounds often shift toward alkaline values and can fall roughly within 7.15–8.9; moreover, wound pH may change dynamically during healing [37]. Taken together, our results—showing a quantitative decrease in equilibrium swelling from pH 5.0 to pH 8.0 while maintaining overall material stability across this range—support the suitability of the collagen matrix for use under variable wound pH conditions and align with published evidence on pH-dependent hydration and functional stability of collagen-containing scaffolds in biomedical applications.

In parallel, the stability of the three-dimensional structure of the sponges was assessed, since they must persist until the completion of the proliferative stage of wound healing, which on average lasts 21 days [38]. During testing, the sponges lost 5% of their weight over 7 days in DMEM medium. After 21 days, degradation was 11%.

### 2.10. In Vitro Breast Carcinoma Modeling

Biomimicry in tissue engineering is an approach in which artificial constructs are created based on principles implemented in living nature. Our goal was a comprehensive verification of the ability of three-dimensional scaffolds from *R. pulmo* jellyfish collagen to mimic the properties of the matrix of biological tissues, in order to use this to restore or replace damaged structures or create an experimental biological model of a pathological process *in vitro*. Collagen-based scaffolds possess excellent biocompatibility and sufficient mechanical properties, so they are widely used in tissue engineering [39,40].

In our study, we constructed a three-dimensional model of human breast carcinoma based on a jellyfish collagen scaffold. Scaffolds are artificially created matrices for cells from various biomaterials, structurally similar to the intercellular substance of human and animal tissues. The internal architecture of the scaffold, the size, number of pores and channels providing internal communications between pores in the cell-engineered construct can influence cell migration, proliferation, and differentiation. The presence of pores in the scaffold structure ensures three-dimensional cell growth with the formation of multiple processes and intercellular contacts with the formation of a dense cell network [41].

**Figure 8:**
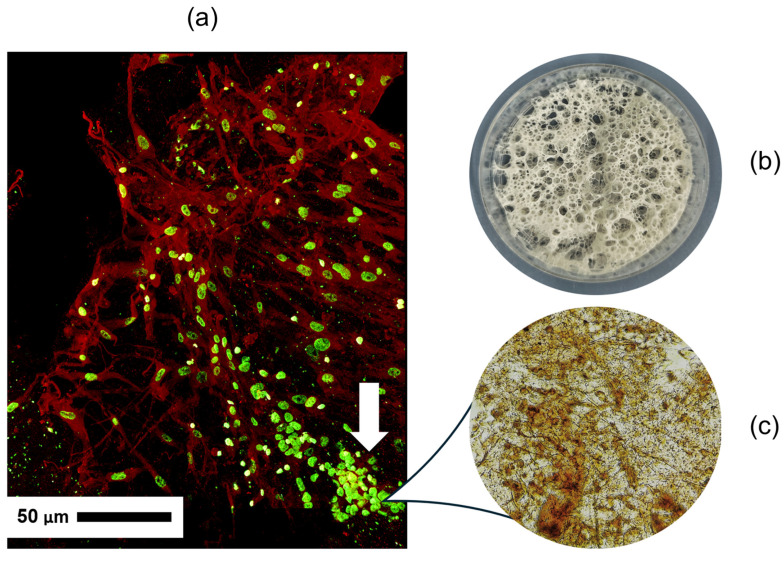
*In vitro* breast carcinoma model constructed on the basis of a scaffold from *R. pulmo* jellyfish collagen: (**a**) confocal laser scanning microscopy demonstrates fibroblasts with red cytoplasm and elongated green nuclei, breast carcinoma cells with round nuclei, white arrow indicates dissemination of breast carcinoma cells from the spheroid (scale bar: 50 μm); (**b**) appearance of the scaffold; (**c**) immunohistochemical analysis shows vimentin formation by cells within the spheroid.

Confocal microscopy data show (Figure 8) that during 5 days of fibroblast cultivation on the surface of the collagen scaffold, cell morphology remained typical, fibroblasts retained the mesenchymal phenotype. Cells adhered to the scaffold material and showed signs of active division. Large cells contributed to active cell migration throughout the entire thickness of the scaffold. On day 5, it was found that fibroblasts successfully colonized the entire scaffold, forming an analog of connective tissue. Spheroids from breast carcinoma cells implanted into the scaffold with fibroblasts after 24 h demonstrate a picture of cell dissemination and invasion into the scaffold tissue.

Thus, jellyfish collagen scaffolds provide conditions for division and colonization of normal human cells and invasion of tumor cells, which corresponds to the biomimicry of type I collagen, which is the main component of the extracellular matrix in both normal and tumor tissues.

Immunohistochemical staining using antibodies to vimentin revealed numerous vimentin-positive cells evenly distributed throughout the thickness of the collagen structure. Brown staining indicates the mesenchymal nature of these cells, indicating the presence of predominantly fibroblast-like elements in the matrix. This is important for confirming the correspondence of the cellular composition to the modeled tissue system.

Thus, during the study, we analyzed the biocompatibility of the collagen matrix obtained from *R. pulmo* jellyfish collagen from the Azov Sea. The uniform distribution of vimentin-positive cells indicates active cell migration or proliferation. It follows that the use of collagen as a biomaterial represents a promising basis for biomedical application.

### 2.11. Implantation of Collagen Sponges in Mice

All animals with implants successfully survived the postoperative period and showed no signs of complications until removal from the experiment. During the study of the obtained histological preparations on the first day after implantation, abundant infiltration of collagen sponges with blood cells was revealed; the observed picture demonstrates the hemostatic effect typical of collagen sponges. In preparations at 21 days, the implant material is covered with a capsule of fibrous connective tissue permeated with implant threads; the relative number of resident cells is 84%, the number of non-resident cells is 16%. The nature of the cellular composition indicates the absence of inflammatory reaction by 21 days and the predominance of fibrocytes and fibroblasts in the capsule structure. The impossibility of determining clear capsule boundaries indicates the completion of integration of the implant into the connective tissue stroma.

**Figure 9:**
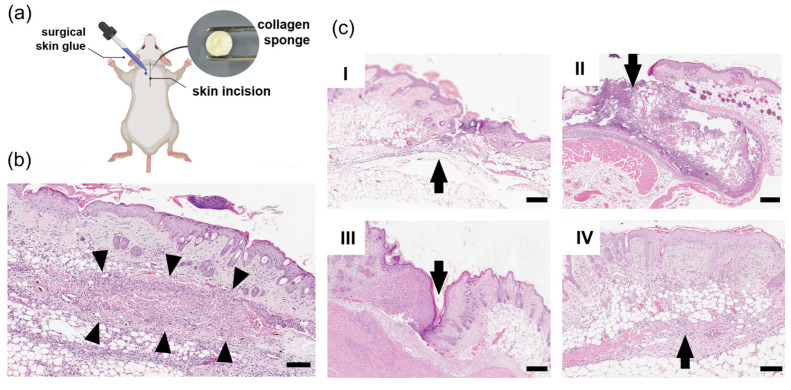
Histological sections of mouse tissues after subcutaneous implantation of collagen sponges. (**a**) Experimental design. (**b**) Representative H&E-stained section of the implantation site at day 21 (scale bar: 1000 μm), triangles indicate the implant area, the fibers of which have been partially resorbed. (**c**) Representative H&E-stained sections illustrating regeneration over time in control mice (no implant) and mice implanted with collagen sponges: I, day 1 post-surgery, control (no implant), arrow indicates the wound (scale bar: 500 μm); II, day 1 post-implantation, arrow indicates the collagen sponge within the wound (scale bar: 500 μm); III, day 21 post-surgery, control (no implant), arrow indicates the scar (scale bar: 1000 μm); IV, day 45 post-implantation, arrow indicates residual sponge fibers at the implantation site (scale bar: 1000 μm). All sections were stained with hematoxylin and eosin (H&E).

Figure 9 presents images of histological preparations of mouse tissues after subcutaneous implantation of collagen sponges.

Forty-five days after implantation, remnants of collagen sponges were detected in the preparations at the implantation site, predominantly replaced by normal connective tissue.

The results showed the hemostatic and long-term anti-inflammatory effect of collagen sponges. Collagen sponges are resorbed within 45 days after implantation through gradual integration into connective tissue, which corresponds to the concept of guided tissue regeneration. The resorption process of the sponges was completed with the formation of vascularized connective tissue.

## 3. Discussion

The present study presents a sequential approach to the isolation, characterization, and primary biological validation of collagen obtained from the jellyfish *R. pulmo*, collected in the Azov Sea, with the aim of assessing its suitability as a marine biomaterial for biomedical applications. Importantly, this work integrates structural, biochemical, cellular, and *in vivo* levels of analysis, which allows not only confirmation of the collagenous nature of the isolated material but also an assessment of its functional relevance in biologically meaningful contexts.

Jellyfish are a valuable source of collagen due to the ease of its extraction, which is related to the primitive structure of the tissues of these organisms [42]. Unlike mammalian connective tissues, jellyfish mesoglea contains relatively low amounts of lipids, pigments, and tightly crosslinked non-collagenous proteins, which simplifies downstream purification and reduces the need for aggressive chemical treatments that may compromise collagen integrity [24,43]. Moreover, jellyfish collagen has been reported to exhibit higher biocompatibility compared to bovine or porcine collagen, including enhanced fibroblast and osteoblast viability as well as a reduced immune response in *in vivo* models [24]. In addition, natural and anthropogenic changes in the natural environment have led to numerous jellyfish population outbreaks in various areas of the Azov Sea, which has caused the beginning of discussions on the problem of harvesting and searching for possible uses of this aquatic organism. Large accumulations of jellyfish not only worsen the oxygen indicator of the reservoir but also reduce the food base of valuable commercial fish species. Organizing large-scale jellyfish harvesting in the Azov Sea for the purpose of further collagen production and products from it would be a solution to the emerging ecological problem [9]. The seasonal nature of jellyfish blooms can be addressed through established preservation strategies: freezing of raw material at −20 °C (as described in Section 4.1), lyophilization of extracted collagen, and storage of lyophilized collagen powder, which remains stable under standard conditions. These approaches enable year-round manufacturing from seasonally collected biomass. The umbrella mass range of 0.5–4 kg in this study reflects the natural size variability of adult *R. pulmo* specimens at the time of collection. At the early stages of the project, jellyfish were additionally sorted by size and collagen was extracted from separate size-based batches; comparison of these batches did not reveal any appreciable differences in the electrophoretic profile or in the basic functional characteristics of the collagen. Using large pooled tissue batches therefore helps to average out individual variability, while each collagen lot is subjected to a standardized quality control panel, including SDS–PAGE and quantitative determination of collagen content with the Sircol™ Insoluble Collagen Assay kit, which allows non-conforming batches to be identified and excluded. Furthermore, the fact that Jellagen Ltd. (UK) produces medical-grade collagen from *R. pulmo* and reports batch-to-batch consistency supports the technical feasibility of standardized collagen production from this species, provided that processing conditions and quality control specifications are rigorously defined and implemented [44].

The use of the acid-soluble extraction approach allowed obtaining collagen with a yield of 26.2%, which indicates the potential of *R. pulmo* as a source of reproducible-yield raw material [2]. It should be noted that the collagen yield from *R. pulmo* may vary depending on the extraction method: the use of pepsin-assisted and/or ultrasound-assisted extraction in other studies has resulted in yields of up to 47% (based on dry weight), highlighting the potential for process optimization [16].

The SDS-PAGE profile with bands in the α-range and high-molecular-weight β/γ-components is consistent with fibrillar collagen-like material and corresponds to published data for *R. pulmo* collagen, including information on the nature of cell adhesion to jellyfish collagen. At the same time, the limitations of SDS-PAGE interpretation for collagens are known (possible “anomalous” mobility and weak expression of individual chains), so electrophoresis should be interpreted as a confirmatory but not exhaustive typing method. We acknowledge that size-exclusion chromatography (SEC) would provide complementary molecular weight distribution data; this analysis is planned for subsequent studies to further refine the characterization of the isolated collagen. Mass spectrometric analysis of amino acid composition is planned as additional characterization methods in future work to obtain a more complete structural profile of the isolated collagen.

Fibrillar features according to TEM data and positive immunolabeling with antibodies to type I collagen additionally support the conclusion about the fibrillar organization of the isolated material [45]. Preservation of a native-like fibrillar ultrastructure is crucial for cell adhesion and function, a characteristic observed in other marine collagen scaffolds used for tissue engineering. Similar ultrastructural characteristics have been reported for other marine collagen-based scaffolds successfully applied in tissue engineering, where maintenance of fibrillar architecture was associated with improved cellular responses [33]. Circular dichroism (CD) spectroscopy is acknowledged as an important tool for confirming triple-helix conformation and will be included in future characterization studies.

The endotoxin level is a critical factor and potential confounder in assessing the biocompatibility of materials, especially for collagen matrices intended for contact with tissues or blood. Endotoxin contamination is known to induce inflammatory and pyrogenic responses that may mask or exaggerate material-related biological effects, thereby complicating biocompatibility assessment [46]. The LAL test results (0.461 EU/µL) support the thesis that the chosen production/processing regime allows achieving low endotoxin load within the calibration range, with author interpretation relative to ISO 10993-11 [47]. However, it should be taken into account that LAL primarily reflects contamination with lipopolysaccharides of Gram-negative bacteria and does not exclude non-endotoxin pyrogens, so when moving toward a medical device, an expanded pyrogenicity/immunoreactivity program is needed.

The absence of detectable cytotoxicity for human fibroblasts (MRC5) in the MTT test and comparable adhesion/proliferation indicators of HeLa cells for jellyfish collagen and rat type I collagen indicate the cytocompatibility of the obtained preparation within the framework of standard *in vitro* screening. These observations are consistent with published data demonstrating that jellyfish collagen in general can support cell adhesion and growth and be applied as a substrate and as a basis for 3D constructs [2].

An important practical result is the production of porous sponges and scaffolds from the isolated collagen, which moves the study from the plane of “biomolecule characterization” to the plane of “functional form-factor matrix.” Transition from molecular characterization to scaffold fabrication is considered a critical step toward translational application of collagen biomaterials [48]. The average pore size (~80 μm) falls within the lower end of the pore size ranges that have been reported to support adequate cell infiltration and mass transport in collagen-based scaffolds, where mean pore sizes of roughly 80–300 μm are generally considered favorable for cell migration and nutrient diffusion [41,49]. Moreover, for skin tissue engineering, collagen/chitosan scaffolds with mean pore sizes in the 100–200 μm range and high porosity have been shown to permit efficient dermal fibroblast infiltration and proliferation, further supporting that pores in the order of tens to a few hundred micrometers are suitable for soft-tissue applications [50]. pH-dependent swelling (greater at pH 5.0 than at pH 8.0) is of interest for wound applications, since the pH of the wound bed can change depending on the stage of healing and chronicity; maintaining matrix integrity under these conditions is an important practical requirement [51].

The observed mass loss in DMEM over 7–21 days indicates partial degradation while maintaining structure at time intervals relevant to early tissue remodeling; however, designing the final product requires more complete degradation kinetics (including in the presence of proteases) and assessment of retention of mechanical properties. The literature on marine collagen and wound materials emphasizes the need for a balance between sorption capacity, structural stability, and controlled biodegradation, which can be regulated by crosslinking and/or compositing [42].

Construction of a 3D breast carcinoma model on a jellyfish collagen scaffold with fibroblasts shows that the matrix supports colonization by stromal cells and dissemination of tumor cells from spheroids into the volume of the construct, which confirms the applicability of the material as a platform for biomimetic *in vitro* modeling [52]. This corresponds to published approaches where marine collagen substrates are used in 2D/3D systems to study cell–matrix interactions in more physiological conditions compared to monolayer culture [52].

Subcutaneous implantation of sponges in mice was accompanied by tolerability without pronounced complications, early signs of implant infiltration with blood cells, and subsequent integration and remodeling over weeks [53]. Wound healing is a staged process. The first stage—hemostasis—begins immediately after tissue damage with aggregation. They release factors that attract leukocytes to the site of damage and promote clot formation, which, in addition to hemostatic action, limits microbial multiplication. Active neutrophil migration in the first hours after damage marks the inflammatory stage [54]. The third, proliferative stage, lasts on average 4 to 21 days from the moment of damage. At this stage, angiogenesis, extracellular matrix synthesis by fibroblasts, and re-epithelialization occur. The appearance of vascularized granulation tissue serves as the basis for repair. Formation of the basement membrane is a key marker signaling the completion of the epithelialization process. The stage following the proliferative remodeling stage can last from 2–3 weeks to several months. During this time, the granulation tissue transforms into a scar consisting mainly of type I collagen. Collagen peptides obtained from *R. pulmo* demonstrated significant potential in wound healing, promoting the production of chemotactic factors and fibroblasts, as shown in this study. Similar studies show that jellyfish and other marine source collagen peptides improve the remodeling phase in wound healing in laboratory mouse skin [55,56,57]. The ability of jellyfish collagen to denature and rapidly degrade at body temperature makes it an ideal candidate for topical application. This feature also minimizes the risk of unexpected immune reactions that may be caused by unidentified components of invertebrate collagen [57].

These observations are consistent with the widely known properties of collagen sponges as hemostatic materials and with data on the applicability of collagen-containing porous constructs for integration into tissues, although the final reaction profile significantly depends on composition, porosity, sterility, and endotoxin load. To strengthen evidence in future studies, it is advisable to add quantitative immunohistochemistry (for example, macrophage polarization markers) and move to direct skin wound models to link subcutaneous biocompatibility with functional endpoints of healing.

The completed set of studies allows us to conclude that marine collagen from jellyfish and products based on it are indeed promising resources for wide application in the medical and bioengineering industry.

Collagen sponges have good biocompatibility, have a long-term anti-inflammatory effect, stimulate connective tissue formation, and can be used for its regeneration.

In conclusion, we note that collagen and collagen/collagen scaffolds represent promising biocompatible and antimicrobial materials for skin tissue engineering with tunable properties that make them suitable for various wound healing tasks.

## 4. Materials and Methods

### 4.1. Collection and Pre-Processing of Jellyfish

One hundred adult jellyfish specimens of *R. pulmo*, Macrì 1778, both males and females, were collected in August 2024 during the seasonal blooming period in the Azov Sea (46°40′ N/37°45′ E, 26–32 °C) by hand. During collection in seawater, tentacles were removed and the intestinal cavity was washed of food residues. Umbrellas weighing from 0.5 to 4 kg were placed in containers with seawater cooled to 8 °C and delivered to the laboratory within 2 h. After delivery, the jellyfish were washed under running cold tap water to remove sand and other contaminants and frozen at −20 °C until further processing.

Pre-processing of raw materials before collagen extraction included three cycles of thawing and freezing for 3 h of fragmented pieces measuring 2 × 2 cm. To remove moisture, non-collagen proteins, pigments, and fats, thawing was conducted in 0.1 M sodium hydroxide (NaOH) solution (LLC. BioloT, St. Petersburg, Russia).

### 4.2. Jellyfish Collagen Extraction

After the final thawing in 0.1 M NaOH, jellyfish tissue fragments were washed in distilled water acidified with 1% by volume HCl (LLC. NevaReaktiv, St. Petersburg, Russia) until pH neutralization. Then, the cooled jellyfish tissues were mixed with 0.5 M acetic acid (LLC. NevaReaktiv, St. Petersburg, Russia) in a ratio of 1:1 (*m*:*m*) and crushed in a blender with short pulses until a homogeneous mass. The obtained homogenate was mixed with 0.5 M acetic acid in a ratio of 1:3 (*v*:*v*). Collagen extraction was carried out for 48 h with constant stirring at 4 °C. After 48 h, the mixture was passed through a sieve with 0.5 mm cells to separate undissolved tissues. Undissolved tissues were re-treated with 0.5 M acetic acid in a ratio of 1:3 (*v*:*v*), stirring the mixture for 1 h at 4 °C, after which it was again passed through the sieve. In total, treatment of undissolved tissues was carried out 3 times. The obtained acid extract was subjected to centrifugation at 10,000 *g* for 1 h at 4 °C to remove the finest insoluble particles, the precipitate of which was removed. The supernatant was collected and collagen was precipitated by adding NaCl (LLC. Diaem, Moscow, Russia) to a final concentration of 0.9 M.

Collagen precipitate was isolated by centrifugation at 10,000 *g* for 1 h at 4 °C. For all subsequent stages, solutions were prepared in deionized pyrogen-free water, and all procedures with collagen and its derivatives were performed aseptically in a laminar flow of a microbiological safety box to ensure sterility. Collagen precipitate was dissolved in 0.5 M acetic acid and the resulting solution was subjected to dialysis using a 12 kDa membrane against 0.1 M, 0.05 M and 0.025 M acetic acid for 24 h, and then against deionized pyrogen-free water. The obtained collagen was either used *ex tempore* for further stages of the study directly as a hydrogel or subjected to lyophilization. Protein concentration was determined using Bradford reagent (K002-500, FineTest, Wuhan, China) in a SpectroSTAR Nano Plate Reader spectrophotometer (BMG Labtech, Ortenberg, Germany) using bovine serum albumin (BSA) (LLC. Diaem, Moscow, Russia) as a standard. Data processing was performed in MARS Data Analysis Software Ver. 3.33 (BMG Labtech, Ortenberg, Germany). Collagen yield (%) was calculated according to the following formula [3]:

*C*_wet_ = *W*_exc_/*W*_wjf_ × 100, (1)

where *C*_wet_—collagen yield (wet) %, *W*_exc_—weight of extracted collagen (g), *W*_wjf_—weight of wet jellyfish (g).

### 4.3. Collagen Extraction from Mouse Tails

As a positive control in this study, acid-soluble type I collagen from mouse tails was used. To obtain it, freshly severed mouse tails were treated three times with 70% ethanol, washed with PBS cooled to 4 °C pH 7.4, and the skin was removed using a scalpel, exposing the tendons. Tendons were pulled from each tail, placing them in ice-cold PBS until the complete collection of material. Then, the tendons were washed in 70% ethanol and 70% isopropanol for 5 min each to remove cellular and lipid material and dried in a laminar air flow. Tendons were cut into pieces 1–2 cm long and placed in 0.5 M acetic acid solution (5 mL per 1 tail). Collagen extraction was performed with constant stirring at 4 °C for 24 h. Then, undissolved tissues were removed by centrifugation at 4 °C, 10,000 *g*, 30 min. The supernatant was subjected to ultrafiltration through a 0.45 μm filter and dialysis using a 12 kDa membrane against 0.1 M acetic acid, 0.05 M and 0.025 M for 24 h, and then against deionized pyrogen-free water.

Protein concentration was determined using Bradford reagent in a SpectroSTAR Nano Plate Reader spectrophotometer (BMG Labtech GmbH, Ortenberg, Germany) using BSA (LLC. Diaem, Moscow, Russia) as a standard. Data processing was performed in MARS Data Analysis Software Ver. 3.33 (BMG Labtech GmbH, Ortenberg, Germany). The obtained collagen was either used *ex tempore* for further stages of the study directly as a hydrogel or subjected to lyophilization.

### 4.4. SDS-Polyacrylamide Gel Electrophoresis (SDS-PAGE)

To assess the type and purity of collagen obtained from *R. pulmo* jellyfish, protein separation in polyacrylamide gel in the presence of sodium dodecyl sulfate according to Laemmli was used. Lyophilized collagen powder was dissolved in 0.5 M acetic acid overnight at 4 °C. To remove the undissolved fraction, samples were centrifuged at 10,000 *g* for 10 min and the supernatant was collected for analysis. The pH of the supernatant was determined and adjusted to normal values. Protein content was determined using Bradford reagent on a SpectroSTAR Nano Plate Reader spectrophotometer using BSA as a standard. Data processing was performed in MARS Data Analysis Software Ver. 3.33 (BMG Labtech GmbH, Ortenberg, Germany).

Samples were mixed with Sample Buffer (62.5 mM Tris-HCl, pH 6.8, 10% glycerol, 2% SDS, 0.01% bromophenol blue, 5% β-mercaptoethanol) in a 1:1 ratio. Then, the samples were incubated for 10 min at 70 °C. Samples containing 25 μg of protein in 15 μL were subjected to electrophoretic separation in 10% polyacrylamide gel in the presence of SDS in mini-PROTEAN Tetra (Bio-Rad Laboratories, Inc., Hercules, CA, USA). Affinity Prestained Protein Ladder (KF8009, Affinity Biosciences Ltd., Changzhou, China) was used as a standard protein marker. Electrophoresis was carried out at 120 V for 20 min, then at 160 V for 1.5 h. The obtained gels were stained with Coomassie blue solution (Coomassie R250 0.25% (LLC. Diaem, Moscow, Russia), ethanol 40%, acetic acid 9%) overnight at 24 °C in the dark. Stained gels were washed repeatedly with distilled water and photographed on a high-resolution gel documentation system SH-Advance523 (Shenhua Science Technology Co., Ltd., Hangzhou, China).

### 4.5. Endotoxin Determination

To determine bacterial endotoxins in the obtained collagen samples, Bioendo™ End-point Chromogenic Endotoxin Test Kit (Xiamen Bioendo Technology Co., Ltd., Xiamen, China) was used according to the manufacturer’s instructions. Test results were determined using a SpectroSTAR Nano Plate Reader spectrophotometer (BMG Labtech GmbH, Ortenberg, Germany); data processing was performed in MARS Data Analysis Software Ver. 3.33 (BMG Labtech GmbH, Ortenberg, Germany).

### 4.6. Transmission Electron Microscopy

Fragments of lyophilized collagen films were fixed in 2.5% glutaraldehyde (Aurion, Wageningen, The Netherlands), washed in PBS, and additionally post-fixed in 1% OsO4 solution (LLC. Reakhim, Moscow, Russia) in phosphate buffer for 1.5 h. Then, the samples were dehydrated in ascending concentrations of alcohols and absolute ethanol, processed in three changes in propylene oxide, and embedded in epoxy resin based on Epon-812. Ultrathin sections were made using an EM UC 7 ultramicrotome (Leica Microsystems, Wetzlar, Germany) and an ultra 45° diamond knife (Diatome AG, Biel, Switzerland). Sections were contrasted with uranyl acetate and lead citrate and examined in a Jem-1011 transmission electron microscope (Jeol Ltd., Tokyo, Japan) with an accelerating voltage of 80 kV.

### 4.7. Immunofluorescence Analysis

For immunofluorescence analysis, collagen hydrogel was applied as a film on a glass slide and dried in a Labconco FreeZone 2.5 L-50 C freeze dryer (Labconco Corp., Kansas City, MO, USA) for 24 h. After drying, a drop of PBS was applied to the film and the preparation was frozen at −20 °C for 20 min. Thawing was performed by applying a drop of PBS at room temperature. The preparation was incubated in a humid chamber in a mixture of primary rabbit polyclonal antibodies to type I collagen Col1A1 (ABclonal Technology, Wuhan, China) at 1:100 dilution for 30 min. Then, the preparation was incubated in a mixture of secondary goat anti-rabbit antibodies Abberior Star Green 488 (Abberior Instruments GmbH, Göttingen, Germany) at 1:200 dilution for 20 min. After that, anti-fade solution (Abberior Instruments GmbH, Göttingen, Germany) was applied to the preparation, covered with a coverslip, and examined using an Abberior Facility Line laser scanning confocal microscope (Abberior Instruments GmbH, Göttingen, Germany) using a 60× objective with oil immersion. Channels: excitation laser 488 nm, detector 498–608 nm.

Images were obtained using iMSPECTOR Image Acquisition & Analysis Software v16.3 (Abberior Instruments GmbH, Göttingen, Germany). As a positive antibody control, a mouse tail collagen preparation prepared similarly was used. PBS was used as a negative control.

### 4.8. Cell Culture

HeLa cell lines, normal human fibroblasts MRC5, and human breast carcinoma BT20 were obtained from the Cell Bank of the National Medical Research Center of Oncology in Rostov-on-Don, Russia. Cells were maintained at 37 °C in DMEM medium (LLC. BioloT, St. Petersburg, Russia), supplemented with 10% fetal calf serum (LLC. BioloT, St. Petersburg, Russia) and 2 mM L-glutamine (LLC. BioloT, St. Petersburg, Russia) in an atmosphere of 5% CO_2_ and 95% humidity. All cell lines were tested for mycoplasma contamination by PCR upon receipt from the biobank and prior to each experiment in accordance with internal standard operating procedures. The following passage ranges (P) were used: HeLa–P5–P15; MRC-5–P5–P10; BT29–P5–P15. Antibiotics and antimycotics were not used when working with cells in this study. To determine cell viability and count live cells, the trypan blue exclusion method (LLC. BioloT, St. Petersburg, Russia) and counting with a Neubauer counting chamber were used.

### 4.9. Cytotoxicity Assessment Using MTT Test

From the stock collagen solution with a concentration of 7.5 mg/mL in 0.1 M acetic acid, a working solution was prepared by mixing 1 mL of stock solution with 14 mL of PBS.

As a control, type I collagen from rat tails (1.2.001. LLC. Biolot, St. Petersburg, Russia) was used, the solution of which was prepared similarly.

Before the analysis, a suspension of normal human fibroblasts MRC5 was added in 200 μL volumes to the wells of a 96-well plate at a concentration of 2000 cells per well and cultured for 24 h in DMEM medium (LLC. BioloT, St. Petersburg, Russia) with 10% fetal calf serum (LLC. BioloT, St. Petersburg, Russia), after which the medium in the wells was replaced and 22 μL of jellyfish collagen working solution was added. As a control, cells in DMEM medium with the addition of 22 μL of solvent not containing collagen (1 mL 0.1 M acetic acid and 14 mL PBS) were used. After 72 h, MTT reagent (LLC. NPP “PanEco”, Moscow, Russia) was added to the experimental wells at a final concentration of 5 mg/mL. After 4 h, the solution was removed from the wells and 100 μL of dimethyl sulfoxide (LLC. BioloT, St. Petersburg, Russia) was added to dissolve formazan crystals. After vigorous shaking of the plate, the optical density in each well was measured using a SpectroSTAR Nano Plate Reader microplate spectrophotometer (BMG Labtech GmbH, Ortenberg, Germany) at wavelengths of 555 and 650 nm. Data processing was performed in MARS Data Analysis Software Ver. 3.33 (BMG Labtech GmbH, Ortenberg, Germany). Cell viability was expressed as a percentage of the total number of viable cells, calculated as the ratio of the average optical density of each sample to the average optical density of the negative control (cells without collagen).

### 4.10. Cell Adhesion and Proliferation Analysis

From the stock collagen solution with a concentration of 8 mg/mL in 0.1 M acetic acid, a working solution was prepared by mixing 1 mL of stock solution with 14 mL of PBS.

Ten ml of working solution was added to Petri dishes (Ø = 90 mm, Sovtech, Novosibirsk, Russia) made of plastic not treated for cultivation and left overnight at 4 °C. Then, the dishes were washed with PBS and saturated with 1% BSA for 60 min. Suspensions of HeLa and MRC5 cells in serum-free DMEM medium (LLC. BioloT, St. Petersburg, Russia) at a concentration of 1 million cells were added to the dishes and incubated for 60 min at 37 °C. Non-adhered cells were removed with the medium, the dish was washed with DPBS without calcium and magnesium, and cells were detached with trypsin-EDTA solution (LLC. BioloT, St. Petersburg, Russia). The cell suspension was stained with trypan blue and concentration was counted using a Neubauer counting chamber.

As a control, type I collagen from rat tails (1.2.001. LLC. Biolot, St. Petersburg, Russia) was used, the solution of which was prepared similarly. Untreated Petri dishes were used as a negative control.

Data were presented as the mean of three replicates, and each experiment was performed at least three times.

### 4.11. Production and Characterization of Collagen Sponges and Scaffolds

#### 4.11.1. Sponge Production

Using freeze-drying, controlled porous three-dimensional structures from collagen were obtained. To obtain sponges, collagen hydrogel with a concentration of 10 mg/mL was cooled to 4 °C, 15 mL of which was placed in a Petri dish (Ø = 60 mm, Sovtech, Novosibirsk, Russia) and frozen at −20 °C for 2 h and at −80 °C overnight, then dried in a Labconco FreeZone 2.5 L-50 C freeze dryer for 24 h. To obtain scaffolds, collagen hydrogel with a concentration of 4 mg/mL was cooled to 4 °C and dried in a Labconco FreeZone 2.5 L-50 C freeze dryer for 24 h without freezing. All stages of sponge and scaffold production were performed aseptically in a laminar flow of a microbiological safety box to ensure sterility; additionally, constructs after lyophilization were treated with UV for 60 min. To obtain sponges in a 96-well plate, 100 μL of hydrogel was added to the wells, after which they were frozen and dried as described above.

#### 4.11.2. Scanning Electron Microscopy

Visualization of the surface of collagen scaffolds was performed using a Crossbeam 340 scanning electron microscope (SEM) (Carl Zeiss Microscopy GmbH, Oberkochen, Germany) using an Everhart-Thornley secondary electron detector (Carl Zeiss Microscopy GmbH, Oberkochen, Germany). The accelerating voltage was 3–6 kV, aperture size—30 μm. To enable scanning electron microscopy, a thin carbon film (~30 nm) was sputtered onto the surface of the samples. This film allowed the sample not to accumulate charge during scanning, thereby ensuring high quality of the obtained images.

#### 4.11.3. Micro-CT

Contrasting of the studied sponge samples was performed by single immersion in a 5% alcoholic solution of Iodine (potassium iodide + ethanol) (LLC. “Tula Pharmaceutical Factory”, Tula, Russia).

For microtomography, an Xradia Versa 520 setup (Carl Zeiss X-ray Microscopy, Inc., Pleasanton, CA, USA) was used. Scanning parameters: 4× objective, X-ray source voltage 80 kV at 6 W power, voxel size 2.0 μm, exposure time 2 s, without filter on the X-ray source. During scanning, the sample rotated 360° around an axis containing the region of interest. During microtomography, 2401 projections of the sample were collected.

XRMReconstructor version 12.0.8086.19558 software (Carl Zeiss X-ray Microscopy, Inc., Pleasanton, CA, USA) was used to reconstruct the projection set into virtual slices with manual adjustment of center shift (Center shift = 53.071) of the focus of the studied object and with application of Gaussian blur (σ = 0.7). Additionally, the beam spectrum was shifted to a higher energy (harder) region (1.00). Drift correction of the tooth during microtomography was performed with the compensating displacements option activated. DICOM format image rendering was performed in VGSTUDIO MAX 3.5 software environment (Hexagon AB, Stockholm, Sweden).

#### 4.11.4. Swelling and Stability

To assess swelling, sponge samples were weighed to determine dry weight (*w*_0_), after which they were placed in PBS and incubated at 37 °C. Every 20 min, the sponges were removed, excess moisture was removed with filter paper, and they were weighed to determine absorbed moisture (*w*_swollen_). Measurements continued until the weight of the sponge scaffolds reached equilibrium. The swelling coefficient (*Q*) was calculated according to the formula [35]:

*Q* = (*w*_swollen_ − *w*_0_)/*w*_0_. (2)

Cultivation stability tests were performed using complete DMEM medium (LLC. BioloT, St. Petersburg, Russia). At specified time intervals (3, 5, 7, 9 days), scaffolds were removed from the medium, frozen at −80 °C, lyophilized, and weighed (*w*_t_). The percentage of weight loss (%) was determined according to the following formula [35]:

*W*_loss_ (%) = ((*w*_0_ − *w*_t_)/*w*_0_) × 100. (3)

#### 4.11.5. In Vitro Cytotoxicity Analysis

Scaffolds lyophilized in plate wells were wetted for 30 min by adding 200 μL of complete DMEM medium (LLC. BioloT, St. Petersburg, Russia), after which the medium residues were removed and MRC5 cells were added to the wells at a concentration of 4000 cells per well and cultured for 24 h (Point 1). Then, another 200 μL of complete DMEM medium (LLC. BioloT, St. Petersburg, Russia) was added to part of the wells and cultured for another 24 h (Point 2). At each point, the MTT test was performed as described in Section 4.9.

Cell viability was expressed as a percentage of the total number of viable cells, calculated as the ratio of the average optical density of each sample to the average optical density of the negative control (cells without scaffolds).

### 4.12. In Vitro Breast Carcinoma Modeling

General preparation for seeding collagen scaffolds: lyophilized scaffolds (Ø = 6 mm, h = 3 mm) in Petri dishes (Sovtech, Novosibirsk, Russia) were immersed in complete DMEM medium (LLC. BioloT, St. Petersburg, Russia) for swelling for 24 h, after which the medium was removed.

#### 4.12.1. Fibroblast Seeding

Ten ml of complete DMEM medium (LLC. BioloT, St. Petersburg, Russia) with MRC5 suspension at a concentration of 5000 cells/mL was added to Petri dishes with prepared scaffolds. Scaffolds with cells were placed in a CO_2_ incubator at 37 °C, 5% CO_2_ and 98% humidity and incubated for 96 h with medium change every 24 h.

#### 4.12.2. Spheroid Production

Spheroids from human breast carcinoma cells were obtained by the hanging drop method [58]. The cell monolayer was detached by treatment with trypsin–EDTA solution followed by neutralization of trypsin with complete DMEM medium (LLC. BioloT, St. Petersburg, Russia), and cells in suspension were counted with a Neubauer counting chamber. Cell concentration was adjusted to 4 × 10^4^ cells/mL, which corresponded to 1000 cells in 25 μL. To create a humidifying chamber, 5 mL of sterile PBS was added to a sterile 60 mm Petri dish (Sovtech, Novosibirsk, Russia). On the inner surface of the Petri dish lid, 25-μL drops of cell suspension were applied, arranging the drops at a sufficient distance from each other to exclude their contact and merging. The lid with drops was carefully inverted drops down and the dish with PBS was covered. The dish was incubated at 37 °C, 5% CO_2_ for 48 h. Upon reaching the required degree of compaction, spheroids were collected from the drops by carefully washing with DMEM medium (LLC. BioloT, St. Petersburg, Russia). The obtained spheroids were implanted by transferring with a pipette tip into a 96 h scaffold with fibroblasts in DMEM medium and incubated for 72 h with medium change every 24 h. After 72 h, the medium was removed and the scaffold was immersed in 4% paraformaldehyde for fixation for 2 h, after which it was subjected to further studies.

#### 4.12.3. Vimentin Formation Analysis

From fixed scaffold fragments in the spheroid implantation area, tangential sections 100 μm thick were made using a VT 1000E vibratome (Leica, Germany). Immunohistochemistry was performed on floating sections. After instant freezing at −80 °C and thawing in phosphate buffer, sections were placed in primary rabbit polyclonal antibodies to vimentin (Dako, Glostrup, Denmark). As secondary antibodies, RTU EnVision/HRP anti-mouse, anti-rabbit conjugated with horseradish peroxidase (Dako, Glostrup, Denmark) were used. Development of immune complexes was performed in a freshly prepared buffer solution with substrate-chromogen—3,3-diaminobenzidine tetrachloride (DAB)/H_2_O_2_. Light optical examination was performed using a DM 2500 microscope (Leica, Wetzlar, Germany) with a built-in DFC 495 camera (Leica, Wetzlar, Germany).

#### 4.12.4. Immunofluorescence Staining and Confocal Laser Scanning Microscopy

From fixed scaffold fragments in the spheroid implantation area, tangential sections 40 μm thick were made using a VT 1000E vibratome (Leica Microsystems GmbH, Wetzlar, Germany). After washing in phosphate buffer, sections were permeabilized in 0.5% Triton X-100 solution (Sigma-Aldrich, St. Louis, MO, USA) for 30 min and then incubated in primary rabbit antibodies to alpha actin (Cloud-Clone, Wuhan, China) 1:100 for 24 h at 4 °C. Then, after washing, sections were incubated in secondary goat-anti-rabbit Abberior Star Red (Abberior Instruments GmbH, Göttingen, Germany) 1:200 and in Sytox Green dye (Invitrogen, Carlsbad, CA, USA). Then, sections were washed in phosphate buffer, mounted on a glass slide in a drop of anti-fade solution (Abberior Instruments GmbH, Göttingen, Germany), covered with a coverslip, and analyzed using an Abberior Facility Line confocal laser microscope (Abberior Instruments GmbH, Göttingen, Germany) using a 60× objective with oil immersion. Channels: excitation laser 488 nm and 561 nm, detector 498–551 nm and 582–692 nm. Images were obtained using iMSPECTOR Image Acquisition & Analysis Software v16.3 (Abberior Instruments GmbH, Göttingen, Germany).

### 4.13. Implantation of Collagen Sponges in Mice

#### 4.13.1. Animals and Anesthesia

Collagen transplantation experiments were performed on 36 male CD-1 mice aged 14 to 15 weeks and weighing 20–25 g. International, national, and institutional regulations on animal care and use were observed.

Animals were kept in standard cages in groups of 6–7 individuals with free access to food and water. Standard conditions were maintained in the animal housing room: 12 light/12 dark cycle, 22–25 °C, air exchange rate of 18 changes per hour.

Mice were anesthetized by intramuscular injection of telazol (mixture of tiletamine hydrochloride and zolazepam hydrochloride (Zoetis US LLC, Parsippany, NJ, USA)) at a dose of 25 mg/kg and xylazine (2% xylazine hydrochloride solution (Interchemie Werken “De Adelaar” B.V., Venray, The Netherlands)) at a dose of 5 mg/kg.

To assess the depth of anesthesia, such indicators as the absence of plantar reflex and reaction to squeezing the membrane between the toes, reduction or absence of muscle tone in the limbs, as well as slowed regular heart rhythm and breathing rhythm were used. During anesthesia and experimental procedures, the following physiological support measures for animals were taken: prevention of eye dryness and corneal damage by placing eye ointment in the conjunctival sac and maintaining temperature using a heated mat.

#### 4.13.2. Implantation Procedure

Subcutaneous implantation of the collagen sponge was performed in mice in the withers area. After reaching the surgical stage of anesthesia, hair in the interscapular area was trimmed, the skin was treated with 70% ethanol followed by treatment with povidone–iodine, and the animal was fixed. In the projection of the withers, a longitudinal skin incision of about 1 cm was made, after which the subcutaneous tissue was separated in the cranio-caudal direction, forming a subcutaneous pocket of sufficient size to accommodate the collagen sponge, while avoiding damage to the underlying muscles. The collagen sponge (Ø = 7 mm, h = 1 mm) was placed in the formed subcutaneous pocket using forceps so that it was positioned flat and did not cause excessive tension of the surrounding tissues. After implantation, the skin wound edges were opposed and the wound was closed with tissue glue by applying a thin continuous layer of glue along the incision line while tightly holding the opposed edges until the glue polymerized. In the control group, animals underwent similar intervention without implantation of the collagen sponge. Upon completion of the operation, animals were placed in a warm box until recovery from anesthesia, the integrity of the glue suture and general condition were monitored, and postoperative analgesia was continued as necessary in accordance with the ethical protocol.

#### 4.13.3. Histological Examination of Tissues

Animals were removed from the experiment on days 1, 3, 5, 7, 21 and 45. Tissues in the surgical intervention area were excised and fixed by immersion in 10% buffered formalin (pH 7.2–7.4). Then, the material was washed in buffer, dehydrated in ascending concentrations of alcohols, and embedded in a paraffin block using a Histostar station (Thermo Fisher Scientific, Waltham, MA, USA). From paraffin blocks, paraffin sections 4 μm thick were made on an HM 340E microtome (Thermo Fisher Scientific, Waltham, MA, USA), which were mounted on slides coated with poly-L-lysine coating and dried. Some sections were stained with hematoxylin and eosin, other sections were used for Masson’s trichrome staining with aniline blue (LLC. Biovitrum, St. Petersburg, Russia). Sections were deparaffinized to distilled water; solutions of Weigert’s iron hematoxylin, alcoholic solution of picric acid, Masson’s acid fuchsin, phosphomolybdic acid, and Masson’s aniline blue were sequentially applied to them according to the kit instructions. Then, sections were dehydrated in ascending concentrations of alcohols, cleared, mounted in balsam, and covered with a coverslip. Light optical examination was performed using a DM 6000 microscope (Leica Microsystems GmbH, Wetzlar, Germany) with a built-in DFC 495 camera (Leica Microsystems GmbH, Wetzlar, Germany).

On microphotographs, the composition of the cell layer in the implant area was assessed. The percentage of connective tissue cells was calculated after counting 100 cells in 10 non-overlapping fields of view. To assess the reaction of connective tissue to the introduction of the implant, the values of the cellular index (ratio of the number of resident cells to the total number of non-resident connective tissue cells) were determined according to the following formula: Cellular index (CI) = resident cells/non-resident cells, where resident cells—total number of macrophages, fibroblasts, and fibrocytes; non-resident cells—total number of granulocytes and monocytes in the cell layer of the peri-implant capsule. At CI = 1, a conclusion was made about the predominance of reparative tendencies. To determine the reliability of differences in means, the confidence interval method was used; differences in mean values were considered statistically significant at *p* ≤ 0.05.

### 4.14. Statistical Analysis

Experiments were performed in 3 biological replicates unless otherwise indicated. For values corresponding to a normal distribution, as assessed by the Shapiro–Wilk test and homogeneity of variances confirmed using the Brown–Forsythe test, statistical significance was evaluated using Student’s *t*-test with Bonferroni correction. Data were processed as mean ± standard error of the mean (SEM), and the level of statistical significance was set at *p* < 0.05 for most statistically processed analyses and at *p* < 0.01 for tests performed on cell cultures.

## 5. Conclusions

This is the first study that evaluates collagen from *R. pulmo* jellyfish collected on the Russian coast of the Azov Sea and conducts a comprehensive study of its mechanical, structural, and biological properties *in vitro* and *in vivo* in a unified sequential bioproduct development process.

Extraction of acid-soluble collagen provided a high yield of material (26.2%), and the results of SDS-PAGE, TEM, and immunolabeling confirmed the collagen nature of the preparation and its similarity to fibrillar type I collagen.

It was shown that the obtained collagen is characterized by low endotoxin load (LAL test: 0.461 EU/µL), which is an important element of quality control for further translation of collagen matrices toward medical devices. In *in vitro* experiments, *R. pulmo* collagen demonstrated cytocompatibility with human cells and provided adhesion and proliferation at a level comparable to mammalian type I collagen, as confirmed by the MTT test on MRC5 and adhesion/proliferation tests on HeLa.

Based on *R. pulmo* collagen, porous 3D sponges/scaffolds were successfully manufactured; microstructural analysis showed an average pore size of about 80 ± 6.2 μm, and swelling and stability tests confirmed the functional properties of the matrix under conditions relevant to culture media and the wound microenvironment. Scaffolds supported 3D cell cultivation and were applicable for constructing an *in vitro* breast carcinoma model, demonstrating matrix biomimicry and suitability for modeling cell invasion.

In *in vivo* tests, subcutaneous implantation of collagen sponges in mice was accompanied by favorable tolerability, early infiltration of the implant with blood cells (a sign of hemostatic behavior), formation of a connective tissue capsule without signs of pronounced inflammation by 21 days, and partial resorption/replacement with connective tissue by 45 days. The totality of results allows considering *R. pulmo* collagen as a promising candidate for replacing terrestrial mammalian-derived collagen in a number of applications, primarily in biomaterial formats for tissue engineering and wound treatment.

In the future, it seems advisable to develop international strategic initiatives for organizing sustainable jellyfish harvesting for the purpose of processing them into valuable natural bioactive compounds for biomedical application.

## Data Availability

The original contributions presented in this study are included in the article. Further inquiries can be directed to the corresponding author.

## Abbreviations

The following abbreviations are used in this manuscript:

BSA	bovine serum albumin
CI	cellular index
DAB	3,3′-diaminobenzidine
DICOM	Digital Imaging and Communications in Medicine
DMEM	Dulbecco’s modified Eagle’s medium
DPBS	Dulbecco’s phosphate-buffered saline
EU	endotoxin units
GOST	State Standard (Gosudarstvennyy standart)
HRP	horseradish peroxidase
ISO	International Organization for Standardization
JCol	jellyfish collagen
kDa	kilodalton(s)
LAL	Limulus amebocyte lysate (assay)
MTT	3-(4,5-dimethylthiazol-2-yl)-2,5-diphenyltetrazolium bromide (assay)
PBS	phosphate-buffered saline
RTU	ready-to-use
SDS	sodium dodecyl sulfate
SDS–PAGE	sodium dodecyl sulfate–polyacrylamide gel electrophoresis
SEM	scanning electron microscopy
TEM	transmission electron microscopy
UV	ultraviolet
XRM	X-ray microscopy
